# Association between sensitivity of viral thymidine kinase-associated acyclovir-resistant herpes simplex virus type 1 and virulence

**DOI:** 10.1186/s12985-017-0728-2

**Published:** 2017-03-21

**Authors:** Natsumi Omura, Hikaru Fujii, Tomoki Yoshikawa, Souichi Yamada, Shizuko Harada, Takuya Inagaki, Miho Shibamura, Haruko Takeyama, Masayuki Saijo

**Affiliations:** 1 Department of Virology 1, National Institute of Infectious Diseases, 1-23-1 Toyama, Shinjuku-ku, Tokyo, 162-8640 Japan; 2 Department of Life Science and Medical Bioscience, Waseda University, 2-2 Wakamatsu-cho, Shinjuku-ku, Tokyo, Japan

**Keywords:** Herpes simplex virus type 1, Acyclovir, Resistance, Thymidine kinase, Virulence

## Abstract

**Background:**

Acyclovir (ACV)-resistant (ACVr) herpes simplex virus type 1 (HSV-1) infections are concern in immunocompromised patients. Most clinical ACVr HSV-1 isolates have mutations in the viral thymidine kinase (vTK) genes. The vTK-associated ACVr HSV-1 shows reduced virulence, but the association between the level of resistance and the virulence of the vTK-associated ACVr HSV-1 is still unclear.

**Methods:**

The virulence in mice of 5 vTK-associated ACVr HSV-1 clones with a variety of ACV sensitivities, when inoculated through intracerebral and corneal routes, was evaluated in comparison with ACV-sensitive (ACVs) parent HSV-1 TAS.

**Results:**

Although all the 5 ACVr HSV-1 clones and ACVs HSV-1 TAS showed a similar single-step growth capacity in vitro, the virulence of ACVr HSV-1 clones significantly decreased. A 50% lethal dose (LD_50_) of each clone was closely correlated with 50% inhibitory concentrations (IC_50_), demonstrating that the higher the ACV-sensitvity, the the higher the virulence among the ACVr clones. One of the ACVr HSV-1 clones with a relatively low IC_50_ value maintained similar virulence to that of the parent TAS. The infection in mice with ACVr HSV-1 due to a single amino acid substitution in vTK induced local diseases, keratitis and dermatitis, while vTK-deficient clone did not.

**Conclusions:**

A statistically significant correlation between the virulence and susceptibility to ACV among ACVr HSV-1 clones was demonstrated.

## Background

Acyclovir (ACV, 2-amino-1,9-dihydro-9-((2-hydroxyethoxy)methyl)-6H-purin-6- one) is an effective first-line antiviral drug to treat herpes simplex virus type 1 (HSV-1) infections. ACV is a guanosine analogue that is monophosphorylated mostly by viral thymidine kinase (vTK), followed by phosphorylation by cellular kinases to become the active form, ACV-triphosphate (ACV-TP). ACV-TP can be incorporated into a viral DNA chain by the action of viral DNA polymerase, resulting in inhibiting viral genome replication by the termination of viral DNA elongation at the site of incorporation, because it does not have 3’-OH in the side chain [1].

ACV-resistant (ACVr) HSV-1 emerges with high frequency in immunocompromised patients [2–4]. Most of clinical ACVr HSV-1 isolates have mutations in the thymidine kinase (TK) genes, and the remaining having mutations in the viral DNA polymerase genes [5–9]. It was reported that vTK was dispensable in cell culture replication; however, it played an important role in inducing virulence in animal models [10–13]. Viral thymidine kinase-deficient HSV-1 impaired viral replication, virulence, establishment of latency, and reactivation in mice [9, 11, 13–17]. Some mutant viruses, which had low TK activity or alter the ability to phosphorylate ACV, showed reduced pathogenicity, but some did not [11, 18, 19]. However, it remains unknown whether there is any relationship between the level of HSV-1 TK activity, susceptibility to ACV, and the level of virulence.

In the present study, the relationship between the phenotypes of ACVr HSV-1 and virulence in mice through intracerebral or corneal inoculations was evaluated.

## Methods

### Cells

African green monkey kidney (Vero) cells were grown at 37 °C with 5% CO_2_ in Dulbecco’s Modified Eagle’s Medium (DMEM) supplemented with 5% fetal bovine serum, 100 U/ml of penicillin and 100 μg/ml of streptomycin (DMEM-5FBS).

### Virus

HSV-1 TAS was used as the wild ACVs clone [20]. ACVr HSV-1 clones generated from HSV-1 TAS in the presence of ACV in the previous study [21] were also used. The vTK gene nucleotide sequence, TK activity, and susceptibility of TAS and these ACVr clones used to ACV was reported previously. One ACVr HSV-1 clone due to frameshift mutation (CL1) was selected as the vTK-deficient and ACVr HSV-1 clone. Furthermore, the ACVr HSV-1 clones (CL18, CL19, CL22, and CL24) with a variety of ACV sensitivities to ACV due to a single amino acid substitution were selected from the set of ACVr HSV-1 clones. When the vTK activity of HSV-1 TAS was defined as 100%, vTK of CL1, CL18, CL19, CL22, and CL24 were <1.0%, 94%, 1.4%, 10%, and 24%, respectively [21]. The susceptibility of each HSV-1 clone to ACV was re-assessed by plaque reduction assay in Vero cells in the present study, as described previously [21]. The viruses were cultured in DMEM supplemented with 2% FBS, 100 U/ml of penicillin and 100 μg/ml of streptomycin (DMEM-2FBS). The nucleotide sequence of vTK gene of all the clones used was also re-determined for confirmation as described previously [21]. Although the data are now shown, nucleotide sequence of DNA polymerase gene of all the clones used was confirmed to be identical to that of HSV-1 TAS.

### In vitro viral replication of ACVr HSV-1

A Vero cell monolayer in the T-12.5 cm^2^ tissue culture flasks was inoculated with each HSV-1 clone at a multiplicity of infection (MOI) of 5 plaque forming unit (pfu)/cell. After 1 hr absorption, the cells were washed with phosphate buffered saline solution (PBS) and then cultured in DMEM-2FBS. At 0, 6, 12, 24, and 48 hr incubation from the end of absorption, the medium and cells were collected and subjected to freeze-thaw. The viral titers were determined on Vero cell monolayers at each time point per three wells in the 24-well tissue culture plates by plaque forming assay. These experiments were performed independently twice.

### Mice

Female BALB/c mice were purchased from SLC Japan (Kurume, Japan). All animal experiments were approved by the Animal Care and Use Committee of the National Institute of Infectious Diseases (NIID) and were carried out in accordance with the approved guidelines.

### Intracerebral inoculation

Three-week-old mice (3 mice per group) were infected intracerebrally with 50 μl of DMEM-2FBS containing a designated amount of each HSV-1 clone. The mice were observed daily and 50% lethal dose (LD_50_) was determined at 14 days post infection (p.i.) by the Reed and Muench method [22]. This experiment was performed independently twice. When each experiment was carried out, the titer of the virus inoculated was confirmed to be the target by back titration with the plaque reduction assay in Vero cells.

### Corneal inoculation

Five-week-old mice (7 mice per group) were intraperitoneally anesthetized by mixture of medetomidine chloride, midazolam, and butorphanol tartrate, and then infected with 5 μl of DMEM-2FBS that contained 1.0 × 10^6^ PFU of each HSV-1 clone through corneal inoculation per each eye [23]. In control group, surparnatant of mock-infected Vero cells were used. The clinical condition was observed daily and the severity level of dermatitis and keratitis was evaluated using a scoring system for 3 weeks under the criteria shown in Table 1 [23–25]. LD_50_ was determined at 21 days p.i. according to the Reed and Muench method [22]. Tears were collected from both eyes for 7 days p.i. using cotton tips, transferred to 1 ml of DMEM-2FBS, and frozen at -80 °C. The samples were thawed, and then infectious virus concentration was determined by plaque forming assay on Vero cells. This experiment was also performed independently twice. When each experiment was carried out, the titer of the virus inoculated was confirmed to be the target by back titration with the plaque forming assay in Vero cells.

**Table 1:** Score standard for the dermatitis and keratitis condition

Score	Dermatitis condition	Keratitis condition
0	Normal	Normal
1	Mild swelling (eyelid)	Neovascularization (the periphery of eyes)
2	Mild swelling (head)	partial opacity
4	Severe fur loss (head)	Bleb formation
5	-	Ulcer, Necrosis

### Statistical analysis

The in vitro replication capacities was assessed among the HSV-1 clonses by Statistical analyses, which were computed by the GraphPad Prism software version 7.01 (GraphPad software, La Jolla, CA). Dunn’s multiple-comparison test was used to compare the in vitro replication capacities and the amount of virus shedding of each clone with that of the parental HSV-1 TAS in the cells infected with or tears collected from mice infected with each clone, respectively. In the analysis, the virus titer was log10 transformed. A significant difference was considered to be present for any p value of <0.05. The relationship between the sensitivities to ACV and LD_50_ of HSV-1 clones were assessed by the Pearson correlation coefficients.

## Results

### Re-characterization of HSV-1 clones used in the present study: sensitivity to ACV and nucleotide sequence of the viral TK gene

The sensitivity of all the HSV-1 clones (ACVs HSV-1 TAS and ACVr HSV-1 clones) used was re-assessed and confirmed that the order in sensitivity to ACV among the HSV-1 clones used was the same as that in the previous study [21]. Furthermore, the nucleotide sequence of the viral TK gene of all the clones used were confirmed to be identical to those determined in the previous study [21].

### In vitro viral replication of ACVr HSV-1

**Fig. 1:**
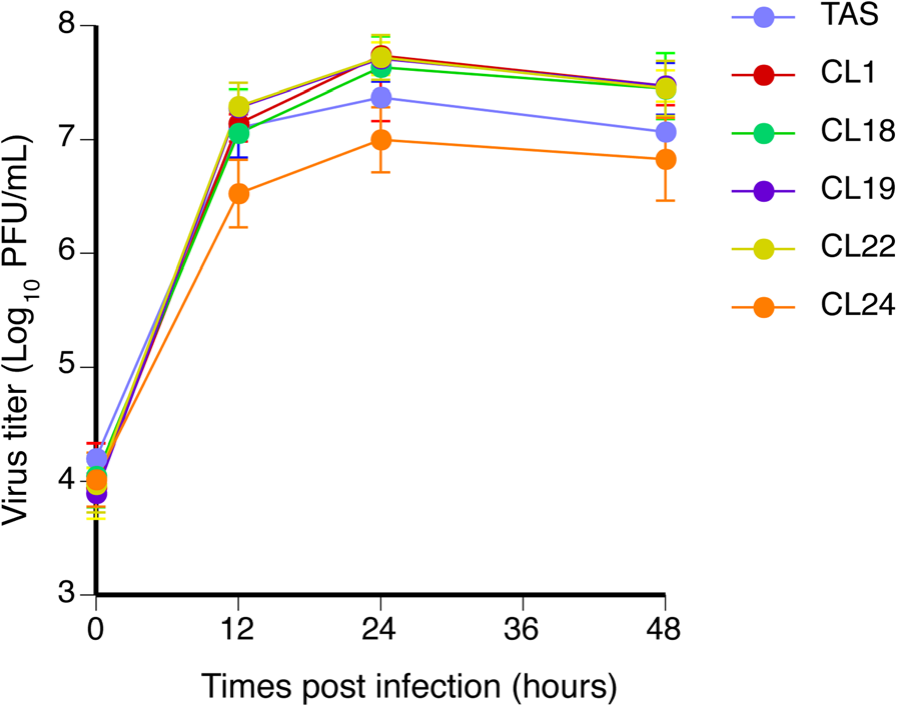
Single-step growth kinetics of all ACVr HSV-1 clones derived from the parent HSV-1 TAS in Vero cells. Vero cells were infected with each of HSV-1 clones at MOI of 5 per cell. The single-step growth kinetics were calculated from two independent experiments

All ACVr HSV-1 clones showed comparable viral replication to that of HSV-1 TAS at each time point in Vero cells (Fig. 1), being consistent with previous studies indicating that vTK activity is not essential for viral replication in tissue culture [13, 17, 26]. There was no statistically significant difference in the replication capacities of each ACVr HSV-1 clone in Vero cells from that of ACVs HSV-1 TAS.

### Virulence of each ACVr HSV-1 clone in mice when inoculated intracerebrally

**Fig. 2:**
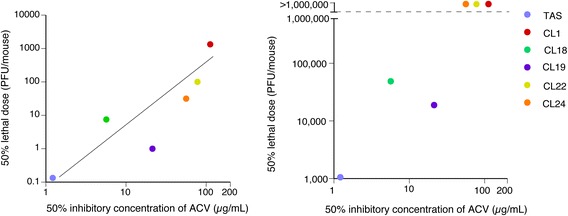
Correlation of the susceptibility to ACV with LD_50_ determined by intracerebral inoculation (**left panel**) and with that determined by corneal inoculation (**right panel**) among the ACVr HSV-1 clones and the parent HSV-1 TAS

The LD_50_ values of ACVr HSV-1 clones were 7.7 × 10^0^ to 1.0 × 10^4^-fold higher than that of HSV-1 TAS when inoculated intracerebrally (Table 2). The intracerebral-LD_50_ was significantly correlated with IC_50_ values of ACV (Fig. 2). The Pearson correlation coefficients (r) and significance values were 0.87 and 0,024, respectively, indiating that the relationship was statistically significant.

**Table 2:** Mutations in vTK gene, susceptibility to ACV, and virulence of HSV-1 clones

HSV-1 clone	Mutations in vTK		Accession number	IC_50_ of ACV (that in the previous study [18]), (μg/ml)	LD_50_ (PFU/head)	
	Nucleotide^a^	Amino acid^a^			Intracerebral inoculation	Corneal inoculation
TAS	No	No	AB047358	1.2 × 10^0^ (6.0 × 10^−1^)	1.3 × 10^−1^	1.1 × 10^3^
CL1	G added within 7G (430–436)		AB047359	1.1 × 10^2^ (>1.0 × 10^2^)	1.3 × 10^3^	>1.0 × 10^6 c^
CL18	C194A	Thr65Asn^b^	AB047372	5.6 × 10^0^ (4.8 × 10^0^)	7.5 × 10^0^	4.9 × 10^4^
CL19	C250T	Pro84Ser	AB047373	2.1 × 10^1^ (1.6 × 10^1^)	1.0 × 10^0^	1.9 × 10^4^
CL22	C734T	Thr245Met	AB047376	7.8 × 10^1^ (8.0 × 10^1^)	1.0 × 10^2^	> 1.0 × 10^6^
CL24	G1007A	Cys336Tyr	AB047378	5.6 × 10^1^ (7.2 × 10^1^)	3.2 × 10^1^	> 1.0 × 10^6^

^a^‘C194A’ represents the nucleotide substitution of cytosine (C) for adenine (A) at position 194

^b^‘Thr65Asn’ represents the amino acid substitution of threonine (Thr) for asparagine (Asn) at position 55

^c^All mice infected with CL1, 22, 24 survived even at an input dose of 10^6^ PFU

### Virulence of each ACVr HSV-1 clone in mice when inoculated through cornea

**Fig. 3:**
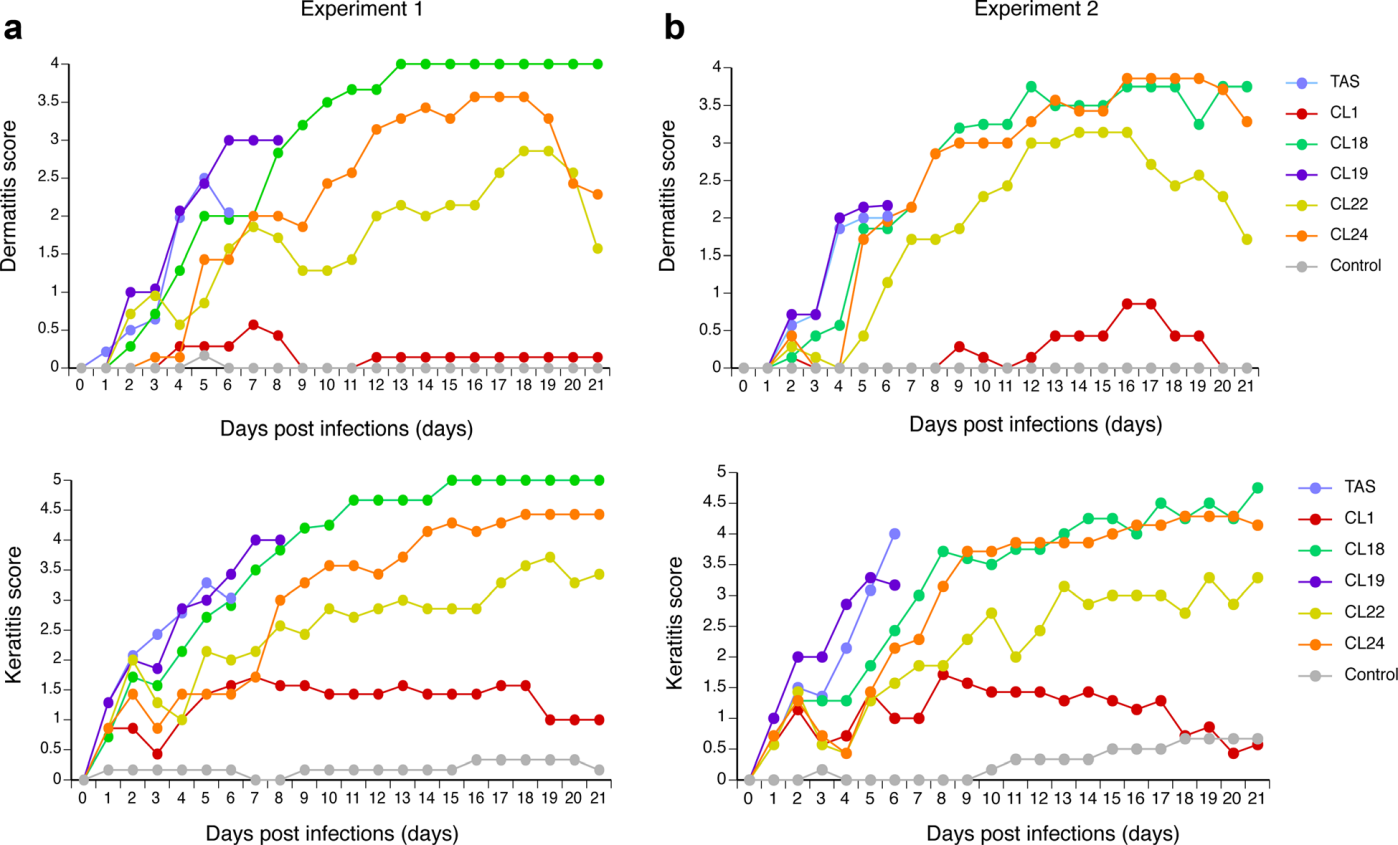
Peripheral pathogenicity of ACVr HSV-1 clones and the parent HSV-1 TAS in mice. Mice were infected with each ACVr HSV-1 clone at the dose of 1.0 × 10^6^ PFU / mouse through corneal route (one eye). This study was conducted twice independently [Experiment 1 (**a**) and Experiment 2 (**b**)]. Severity levels of dermatitis (*upper panels*) and keratitis (*lower panels*) were measured by using the scoring system introduced. All the mice inoculated with TAS or CL19 died within 7 days post infection

The corneal-LD_50_ value of HSV-1 TAS was the lowest among the HSV-1 clones tested, followed by the CL19 and CL18. The corneal-LD_50_ of HSV-1 CL1, CL22, and CL24 were > 1.0 × 10^6^. The corneal LD_50_ of HSV-1 of the CL 19 and CL18 was approximately 1.7 × 10^1^ and 4.5 × 10^1^ times higher than that of HSV-1 TAS, respectively (Table 2, Fig. 2right panel). All the mice inoculated with HSV-1 TAS at the dose of 1.0 × 10^6^ PFU caused lethal infections within 7 days post infection (Fig. 3). All the mice inoculated with CL19 at the same dose also died within 7 days post infection (Fig. 3). Four of all 7 mice and 3 of all 7 mice inoculated with CL18 in Experiment 1 and Experiment 2, respectively (Fig. 3), died between 7 and 11 days post infection.

The mice inoculated ocularly with TAS or CL19 died before 7 days post infection, therefore the regional ocular lesions after 7 days post infection could not be observed. The post 7-days lesions worsend in mice inoculated with ACVr HSV-1 CL18, CL19, or CL 22, while those of the mice inoculated ocularly with CL1 was alsomt the same level as that of the control (Fig. 3).

### Viral shedding in cornea of ocularly infected mice

**Fig. 4:**
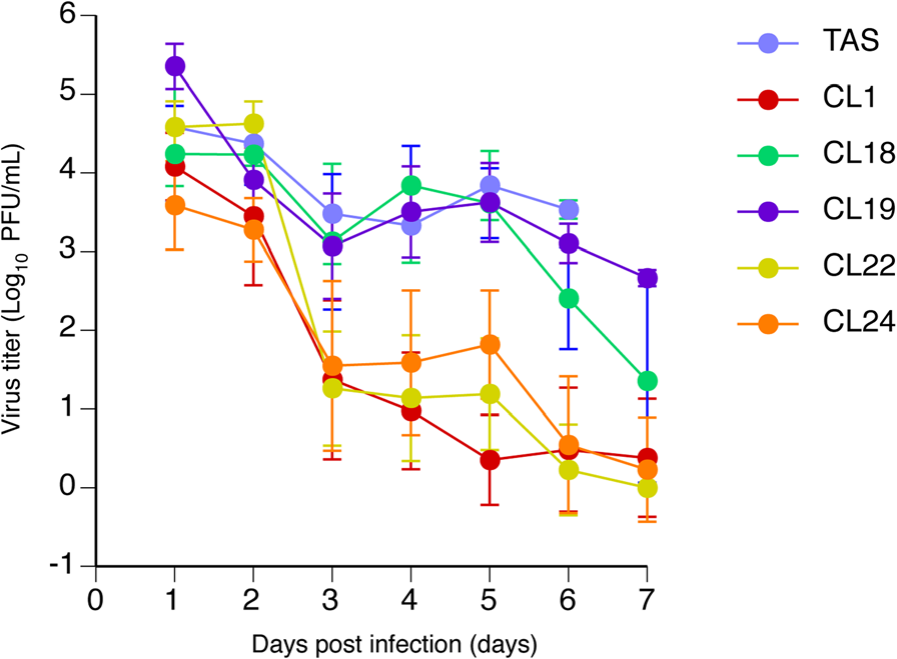
The viral titers in tears of mice inoculated with each virus clone to cornea. Mice were infected with each virus clone at the dose of 1 × 10^6^ PFU through corneal inoculation. Tears were collected every day for 7-day observation period. Viral titer in tears were determined by plaque forming assay in Vero cells. All the mice inoculated with HSV-1 TAS or CL19 died within 6 and 7 days p.i., respectively

The viral loads in tears of mice infected with each clone were almost the same level among all the mouse group within 2 days post infection. However, those of the mice infected with HSV-1 TAS, CL18 or CL19 at the dose of 1.0 × 10^6^ PFU/eye maintained a higher level for 7 days, while those of the mice infected with HSV-1 CL1, CL22 or CL24 showed a obvious decrease in viral loads, particularly after 3 days post infection (Fig. 4). Statistical analyses with Dunn’s multiple-comparison test confirmed the results described above, because the statistically significant decrease in virus titers in tears of the mice infected with each clone against those infected with HSV-1 TAS was demonstrated in CL1-, CL22-, and CL24-infecting mice on days 2–5, days 3–5, and days 1–5, respectively. There was no statistically significant decrease in the titers was demonstrated between HSV-1 TAS-infecting mice and those infected with CL18 or CL19 except for CL19 on day 1.

## Discussion

The results in the present study indicated that vTK-associated ACVr mutants showed lower virulence and pathogenicity as reported previously [11, 12, 14–17]. However, the mechanism of the reduced virulence of the vTK-associated ACVr HSV-1 was not simple in terms of the level of virulence and replication capacity, which were influenced by the route and method of infection, in vivo.

The ACVr mutants with relatively higher susceptibility to ACV retained higher virulence (Figs. 2 and 3), although there was no significant relationship between intracerebral-LD_50_ and vTK activity (Table 2). It indicates that the virulence and pathogenicities are not simply associated with the vTK activity level. In fact, CL19, whose vTK activity was only 1.4% to that of HSV-1 TAS, was more virulent than CL18, whose vTK activity was 94% to that of HSV-1 TAS, when inoculated both through intracerebral and corneal routes (Figs. 2 and 3) [21]. It was reported that vTK-associated ACVr HSV-1 mutant with high vTK activity retained a similar virulence to the parent HSV-1, when inoculated intracerebrally [11]. Harris et al reported that the pathogenicity and the reactivation capability of the majority of vTK-associated ACVr HSV-1 clinical isolates demonstrated low neurovirulence [12]. A unique vTK-deficient ACVr HSV-1 due to a double G insertion in the 7 G-homopolymer stretch in vTK gene showed a relatively higer virulence and reactivation capabiligy from letencey [12, 19]. The mechanism behind the higher pathogenicity was to due to an additional single G-insertion into the G-homopolymer stretch, resulting in the restoration of the vTK open reading frame in vivo [19]. These results suggest that the characteristics of the vTK-associated ACVr HSV-1 in terms of the mutations in the vTK gene and fitness in vivo are the factors for the virulence. Another explanation for the discrepancy between the vTK activity and the virulence might be the method of measuring vTK activities. The vTK activities were measured using the 143B/TK- neo R cell extracts infected with each clone without purification of the vTK protein [21], as the vTK activities were measured for the cell extracts infected with each HSV-1 [11–13, 15], while they were measured for the purified vTK of each HSV-1 [8].

Similar discussion is required for CL24, whose vTK activity maintained about one-fourth of the vTK activity of HSV-1 TAS, showed higher attenuation than that of CL19, whose vTK activity was only 1.4% to that of HSV-1 TAS. To confirm the results that CL19 was more virulent than CL18 and that CL24 showed higher attenuation than CL19 should be studied with using the recombinant HSV-1 with each mutation in vTK genetically engineered.

When the peripheral (corneal) virulence of the ACVr HSV-1 clones were measured, it was demonstrated that the ACVr HSV-1 clones with some degree of vTK activities (CL18, CL22, and CL24) induced local lesions, but the vTK-deficient CL1 did not as reported previously (Fig. 3) [27]. The results suggest that vTK-associated ACVr HSV-1 due to a single amino acid substitution remained peripheral virulence in mice. Further study is needed to clarify whether the peripheral virulence of the single amino acid-substitution based ACVr HSV-1 maintained in humans or not.

To evaluate the replication capacity of vTK-associated ACVr HSV-1 at the site (cornea) of virus inoculation, virus titers in tears of mice inoculated with each of HSV-1 clones through corneal inoculation were measured (Fig. 4). The virus load kinetics in tears of HSV-1 CL18 and CL19 were almost the same as that of HSV-1 TAS (Fig. 4). The mechanism behind the difference in virus load kinetics between relatively less ACVr HSV-1 (CL18 and CL19) inclusing HSV-1 TAS and relatively higly ACVr clones (CL22, CL24, and CL1) can be discussed as follows. All the HSV-1 replicated at the site of infection with the same replication capacity until day 2 post infection. The virus replicated at the site would be transported to ganglions from the inoculation sites, and the virus transported to trigeminal ganglion might replicate in the ganglions [24]. The virus replicated in trigeminal ganglion might be then transported back to peripheral tissues or forward to the brain. The mechanism of high virus titer-maintenance in tears of mice inoculated with TAS, CL18, or CL19 from day 3 to day 7 might be due to the high replication capacity at the local site of inoculation and/or high replication capacity in trigeminal ganglia with an ategrade return of the virus replicated in trigeminal ganglia. It is speculated that the relatively higly ACVr clones (CL22, CL24, and CL1) lacks or have reduced replication capacity in trigeminal ganglia. It was reported that a vTK-deficient HSV-1, which should be ACVr, showed a significantly reduced replication capacity in trigeminal ganglia of mice [28]. This result suggests that CL1 did not replicate in the trigeminal ganglia, when mice were inoculated with CL1 through corneal route. Therefore, the CL1 titer in tears collected from mice corneally inoculated with CL1 decreased after day 3 post infection (Fig. 4). However, the kinetics of the partially vTK-positive CL22 and CL24 were the same kinetic as that of CL1 (Fig. 4). These results also suggest that the replication capacities of vTK-associated ACVr HSV-1 are not regulated solely by the vTK activitiy of the vTK-associated ACVr HSV-1. The replication capacity in trigeminal ganglia of each clone should be assessed to support this speculation.

One of the limitations in this study is that the number of HSV-1 clones (one ACVs HSV-1 and 5 ACVr HSV-1 clones) used was small. Therefore, there is a room for discussion that the conclusion, “The higher the level of ACV-resistance, the lower the virulence in mice”, is still suggestive. Although the number of ACVr HSV-1 clones seems to be small, the sensitivity of these 5 ACVr HSV-1 and ACVs HSV-1 TAS to ACV varied, ranging from approximately 1 μg/ml to over 100 μg/ml. These clones with a variety of ACV-sensitivity were selected from those generated in the previous study [21]. To our knowledge, there have been no reports, in which virulence of several vTK-associated ACVr HSV-1 clones was assessed with ACV-sensitivity, mutations in the vTK, and vTK activities. Therefore, the number of ACVr HSV-1 clones (5) used in this single study is considered acceptable for obtaining the conclusions. Second limitation is that the capacity of replication and establishment of latency in trigeminal ganglia for each clone was not evaluated. To clarify the mechanism behind the difference in virulence (disease severities) among these clones, the capacity of replication and establishment of latency in trigeminal ganglia for each clone should be evaluated in detail. Third limitation in this study might be that recombinant HSV-1 with each amino acid mutation in the vTK gene genetically engineered was not included to exclude the possibility that other factors except for vTK mutations contributed to the difference in the virulence.

## Conclusions

There was a statistically significant correlation between the virulence and the susceptibility to ACV among ACVr HSV-1 clones, suggesting the higher the sensitivity to ACV, the higher the virulence. Single amino acid substitution-based vTK-associated ACVr HSV-1 infection induced local skin lesions, dermatitis and keratitis, in mice, while completely vTK-deficient ACVr HSV-1 did not.

## Abbreviations

HSV-1: Herpes simplex virus type 1
IC_50_: 50% inhibitory concentrations
LD_50_: 50% lethal dose
vTK: viral thymidine kinase
